# Comparison of homocysteine, vitamin B12 and folic acid between rural and urban ageing Indians and its association with mild cognitive impairment and cardiovascular risk factors: a cross-sectional analysis

**DOI:** 10.1093/braincomms/fcae343

**Published:** 2024-10-15

**Authors:** Divya N Mallikarjun, Palash Kumar Malo, Abhishek Mensegere, Ajith Partha, Jonas S Sundarakumar, Thomas Gregor Issac, Latha Diwakar

**Affiliations:** Centre for Brain Research, Indian Institute of Science, Bangalore 560012, Karnataka, India; Centre for Brain Research, Indian Institute of Science, Bangalore 560012, Karnataka, India; Centre for Brain Research, Indian Institute of Science, Bangalore 560012, Karnataka, India; Centre for Brain Research, Indian Institute of Science, Bangalore 560012, Karnataka, India; Centre for Brain Research, Indian Institute of Science, Bangalore 560012, Karnataka, India; Centre for Brain Research, Indian Institute of Science, Bangalore 560012, Karnataka, India; Centre for Brain Research, Indian Institute of Science, Bangalore 560012, Karnataka, India

**Keywords:** homocysteine, mild cognitive impairment, Framingham risk scores, urban India, rural India

## Abstract

The relationship between blood levels of homocysteine (HCY), vitamin B12, folic acid and cognitive impairment is inconclusive. Since HCY is an independent risk factor for cardiovascular diseases, understanding its association with Framingham risk score (FRS) may provide insight into the shared underlying mechanism between cardiovascular disease and cognitive impairment. Cross-sectional analyses utilized baseline data from two ongoing longitudinal studies: the Tata Longitudinal Study of Ageing (*n* = 923), an urban cohort, and Srinivaspura Ageing, NeuroSenescence and COGnition (*n* = 4239), a rural cohort. The study compared the HCY, vitamin B12 and folic acid levels across cohorts and normal versus mild cognitive impairment (MCI) participants. The association between HCY and cognitive status was established using regression models. Three models were analysed: model 1—unadjusted; model 2—adjusted for age, gender, smoking, alcohol consumption, diet, hypertension, cardiac illness, diabetes; and model 3—adjusted for variables in model 2 plus vitamin B12 and folic acid. Correlation was calculated between HCY and FRS. The urban cohort exhibited a significantly higher level of HCY [median (IQR) (17.70 (10.2) versus 14.70 (9.7); *P* < 0.001)], vitamin B12 (251 (231) versus 219 (138); *P* < 0.001) and folic acid (8.21 (8) versus 5.48 (4); *P* < 0.001) levels compared to rural cohort. HCY, vitamin B12 and folic acid levels did not differ significantly between normal and MCI participants in the urban cohort. In the rural cohort, among the age-gender matched MCI-normal, participants with normal cognition had higher levels of vitamin B12 (≥60 years) [227 (152) versus 217 (175); *P* = 0.03] and folic acid (<60 years) [5.91 (4) versus 5.40 (4); *P* = 0.04] compared to MCI. There was no association between HCY and cognitive status in both the cohorts, but there was a significant positive relationship between vitamin B12 deficiency and Clinical Dementia Rating—Sum of the Boxes (CDR-SOB), as well as folic acid deficiency and CDR-SOB in rural and urban cohorts, respectively, within a specific age group. A significant correlation was observed between FRS and HCY in the rural cohort (r = 0.17, *P* < 0.001), but not in the urban cohort. This study revealed significant differences in HCY, vitamin B12 and folic acid levels between the cohorts. In the rural cohort, participants with MCI had lower vitamin B12 and folic acid levels in a certain age group. Association between HCY and cognitive status was insignificant in both the cohorts. A small significant correlation between FRS and HCY was seen in the rural cohort.

## Teaser

Mallikarjun *el al.* investigated the relationship between homocysteine, vitamin B12, folic acid and cognition across urban and rural cohorts. Urban participants had higher homocysteine, vitamin B12 and folic acid levels. No significant association found between homocysteine and cognition. A correlation between homocysteine and Framingham risk score was observed in the rural cohort.

## Graphical Abstract

**Graphical Abstract:**
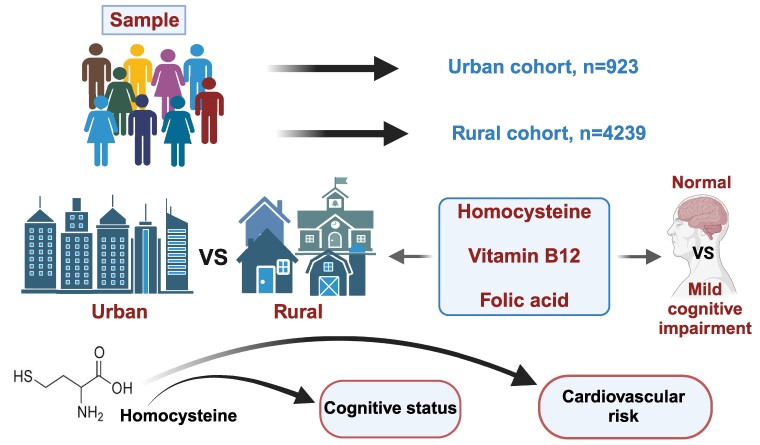

## Introduction

Mild cognitive impairment (MCI) is a transitional phase between normal age-related cognitive decline and dementia.^1^ The prevalence of MCI is steadily increasing in India and has been reported to be 26.1%, according to a previous study.^2^ The higher prevalence of MCI compared to dementia suggests that a significant proportion of people are in the transitional phase, experiencing slight cognitive impairment, but not meeting the criteria for dementia yet. Therefore, identifying the modifiable risk factors associated with MCI could help in preventing its conversion to dementia and thereby reducing the overall dementia burden.

Homocysteine (HCY) is an amino acid produced in the body as a by-product of methionine metabolism. Hyperhomocysteinemia, a condition characterized by elevated level of HCY in blood, is diagnosed when the concentration of HCY in the blood exceeds a normal range (15 µmol/L^3^). Vitamin B12 and folic acid are essential water soluble vitamins that play crucial roles in various physiological processes, including HCY metabolism and cognitive function. A complex relationship exists between HCY, vitamin B12, folic acid, and cognitive impairment. There is a growing interest in understanding this complex relation in the recent years.

Elevated levels of HCY have been associated with increased risk of cardiovascular diseases (CVD),^4^ and evidence suggests that it may promote atherosclerosis, endothelial dysfunction and thrombosis. These vascular changes may reduce the blood flow to the brain, thereby increasing the risk of cognitive impairment and dementia. Numerous studies have examined the impact of HCY, vitamin B12 and folic acid on cognition, yet findings remain inconclusive.^5-7^ While some studies have demonstrated associations between these factors, others have not.

The Framingham risk score (FRS) is an established tool for estimating an individual's 10-year risk of developing CVD.^8^ While FRS provides a comprehensive assessment of CVD risk based on traditional risk factors, HCY serves as an additional biomarker in determining the individual's cardiovascular risk.^9^ Together, FRS and HCY assessment could help identify and thereby reduce the risk of CVD and cognitive impairment, as both have an overlapping risk profile.

Our research comprises prospective, ageing cohort studies conducted in the rural and urban communities of Karnataka, a southern Indian state.^10^ The urban study, known as Tata Longitudinal Study on Aging (CBR-TLSA), is conducted in the metropolitan city of Bangalore. By contrast, the rural counterpart is named Srinivaspura Ageing, Neuro Senescence and COGnition (CBR-SANSCOG) and is conducted in the villages of Srinivaspura, a taluk of Kolar District. The primary goal of these studies is to study the trajectories of cognitive ageing in urban and rural areas, as well as to identify protective and risk factors associated with dementia. Both studies recruit participants who are aged 45 years and above, who are evaluated with detailed multimodal (clinical, cognitive, biochemical, genetic and imaging) assessments, with periodic, long term follow-up. Considering the difference in access to healthcare, occupational types and food patterns between urban and rural settings, it is important to investigate the association between HCY, vitamin B12 and folic acid and cognition in both urban and rural cohorts. These factors could influence the levels of HCY, vitamin B12 and folic acid, as well as their distinct association with CVD and cognition.

The current study was aimed at a) comparing the levels of HCY, vitamin B12 and folic acid between individuals from the urban and rural cohorts, b) comparing the levels of HCY, vitamin B12 and folic acid levels among normal and MCI participants and c) investigate the potential correlation between HCY levels and FRS in the urban and rural cohorts, thus shedding light on the relationship between cardiovascular risk and HCY. The novelty of this study lies in the comprehensive understanding of the interplay between HCY, CVD and cognitive impairment in distinct settings—urban and rural, which in turn can help develop targeted interventions to address the specific needs of each population.

## Materials and methods

### Study population

**Figure 1:**
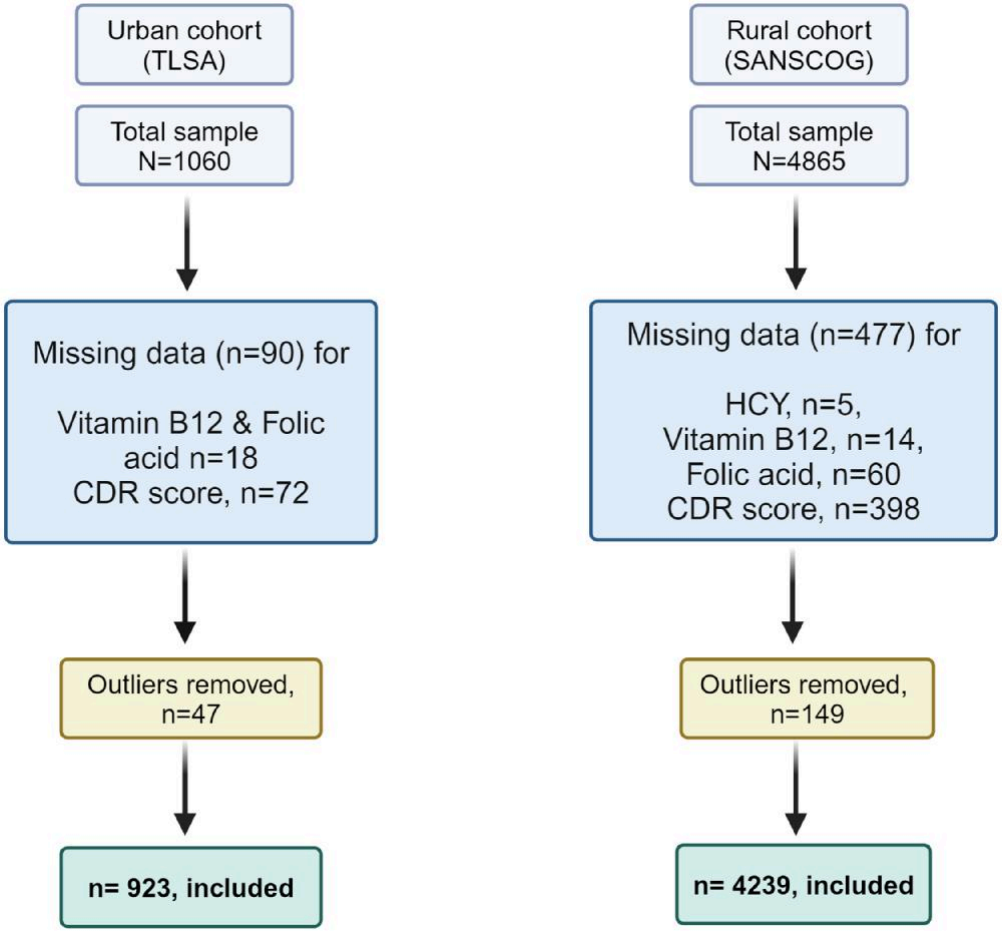
**Flowchart of participants. Flowchart of the participants included in the analysis.** Tata Longitudinal Study on Aging (TLSA); Srinivaspura Ageing, NeuroSenescence and COGnition (SANSCOG); homocysteine (HCY); clinical dementia rating (CDR).

Among the initial 1060 participants in urban and 4865 in rural cohort, 137 and 626, respectively, were excluded from analysis. These exclusions were primarily due to missing data in variables, such as HCY (urban 0%, rural 0.1%), vitamin B12 (urban 1.7%, rural 0.29%), folic acid (urban 1.7%, rural 1.23%) and CDR score (urban 6.8%, rural 8.18%). There were no missing values for the demographic, lifestyle and health-related variables. The reasons for missingness varied from data entry errors to participants declining due to time constraints. Additionally, *n* = 47 outliers were removed from the urban cohort, and *n* = 149 were removed from the rural cohort. Consequently, the final analyses included 923 and 4239 participants in urban and rural cohorts, respectively, who had completed baseline clinical, cognitive assessments and blood investigations (Fig. 1). All the data for urban cohorts were collected between July 2015 and October 2022 and between June 2018 and November 2022 for the rural cohort.

### Inclusion and exclusion criteria

#### Inclusion criteria

1. Participants aged 45 years and above;
2. Residents of Bangalore and Srinivaspura from CBR-TLSA and CBR-SANSCOG cohorts, respectively; and
3. Participants with Clinical Dementia Rating (CDR) global scores of 0 and 0.5.

#### Exclusion criteria

1. Participants diagnosed with dementia or CDR global score ≥ 1;
2. Severe medical condition (e.g. acute myocardial infarction, infective endocarditis, acute coronary condition, active malignancy, end stage of renal disease, etc.) and neurological illnesses (Parkinson's disease, stroke etc.) or those conditions which limit our assessments; and
3. Severe hearing and visual impairment, which interferes with the assessments in the study.

#### Ethics and consent

Both CBR-TLSA and CBR-SANSCOG studies have obtained ethical clearance from the Institutional Ethics Committee (Institutional Review Board) of the Centre for Brain Research, Indian Institute of Science, India. Written informed consent was obtained from all participants for all the assessments, including blood investigations.

### Sample collection

Accordingly, 15 mL of peripheral venous blood was taken after an overnight fasting for various biochemical tests. Blood samples were collected at the subject's home in urban cohort and at the blood collection camps in the rural cohort. Blood samples for serum HCY, vitamin B12 and folic acid analysis were collected in a gel tube. All investigations were performed in NABL-accredited (National Accreditation Board for Testing and Calibration Laboratories) laboratories following the principles of Good Laboratory Practice.

### Biochemical analysis

HCY was measured through the chemiluminescence method, while vitamin B12 and folic acid were analysed with electrochemiluminescence binding assay method using Cobas e 801 immunoassay analyser and Vitros ECI analysers.

For HCY, ≤ 15 µmol/L was considered as normal, and >15 µmol/L as elevated. For vitamin B12, ≥ 200 pg/mL was considered as normal, and <200 pg/mL as deficiency. For folic acid, ≥ 3 ng/mL was considered as normal and <3 ng/mL as deficiency based on the laboratory reference ranges.

### Assessment of MCI

CDR is a global rating scale to diagnose the severity of dementia. It assesses six domains (Memory, Orientation, Judgment and Problem-solving, Community affairs, Home and Hobbies, as well as Personal care), and the total scores range from 0 to 3. For the present study, we used CDR global score for categorizing the study participants into normal (CDR = 0) and MCI (CDR = 0.5).^11^ Additionally, analysis was also done using CDR sum of the boxes (CDR-SOB), with CDR-SOB score of 0 as normal and score of 0.5 to 4 as MCI.^12^

### Assessment of demographic and other variables

Data on demographic variables (age, current gender—female or male), lifestyle factors (diet, alcohol consumption and smoking) and disease history were collected during the clinical assessments through face-to-face interview using standardized questionnaire. Participants were categorized as either following vegetarian or mixed diet, determined by responses to a dietary questionnaire administered, and into never smoked, ex-smokers and current smokers based on smoking status, and as non-drinkers and current drinkers based on alcohol consumption history.

Disease history is based on self-reported and considering clinical findings, as well as blood investigations. Hypertension was taken as those who are on antihypertensive medicines and/or SBP ≥ 140 mmHg or DBP ≥ 90 mmHg.^13^ Diabetes mellitus was taken as the use of oral antidiabetic drugs or insulin and/or fasting sugar ≥ 126 mg/dL, HbA1C ≥ 6.5%.^14^

### Calculation of FRS

FRS was calculated separately for males and females by considering age, high density lipoprotein (HDL), total cholesterol, systolic blood pressure, smoking status and diabetes.^8^

### Statistical analysis

Median (interquartile range, IQR) and frequencies (percentages) are reported for continuous and categorical variables, respectively. Normality was checked for the continuous variables using Shapiro–Wilk test. Mann–Whitney U test or independent *t*-test was used for the continuous variables, and Chi-squared test was used for the categorical variables. We also checked for Box–Cox transformation and used the age-residualized variables in the nonparametric analyses whenever the transformed variables remained skewed after the transformation. Moreover, one-way analysis of variance (ANOVA) or Kruskal–Wallis H test was used for multiple groups, wherein Bonferroni corrected *P*-values were used for multiple comparisons. We also reported Cohen’s d and its 95% confidence intervals (CI) as the effect size wherever applicable. The effect sizes of 0.2, 0.5 and 0.8 were considered as small, medium and large, respectively.^15^

A general linear model (GLM) was used to establish a linear relationship between biomarker levels (HCY, vitamin B12 and folic acid) and socio-demographic lifestyle factors and health conditions to explore diet choice in two cohorts. CDR-SOB was treated as continuous variable. Further, for the dichotomous outcome variables, a binary logistic regression was adopted to estimate the odds ratios (ORs) and its 95% confidence interval (CI) for HCY (≤15 μmol/L and > 15 μmol/L), vitamin B12 (≥200 pg/mL and <200 pg/mL) and cognitive status (normal versus MCI). Three models were analysed: model 1 was unadjusted; model 2 was adjusted for age, gender, smoking, alcohol consumption, diet, hypertension, cardiac illness, diabetes; and model 3 was adjusted for variables in model 2 plus vitamin B12 and folic acid. Additional analyses were done to establish the relationship between different combinations of HCY-vitamin B12 levels and cognition. Furthermore, mediation analyses were used to check whether hypertension, diabetes, cardiac illness and FRS were the mediators or not between HCY and cognition. A bootstrap sample of size 5000 was used to estimate the indirect effect of the mediator and to generate 95% bootstrap CI. We also performed moderation analysis to check whether gender has a moderating effect between HCY and cognition. Spearman correlation coefficient was used to calculate correlation between HCY and FRS. Values less than 2.5 percentile and above 97.5 percentile were considered as outliers and were removed before proceeding with the above analyses. Pair-wise deletion method was adopted for missing data in all the analysis. Statistical significance was checked at 5% level of significance. All statistical analyses were performed using IBM SPSS statistical software version 28.0 (Armonk, NY; IBM Corp. Released 2021),except for the mediation analyses which were performed using Stata version 18 (StataCorp. 2023. Stata Statistical Software: Release 18. College Station, TX: StataCorp LLC).

## Results

Table 1 summarizes the demographic characteristics of participants in urban and rural cohorts. Median age of the participants was 64 (14) and 58 (15) years in the urban and rural cohorts, respectively. Accordingly, 48.4% in the urban cohort and 52.3% in the rural cohort were females. Majority of them in the urban and rural cohorts were non-smokers and non-alcoholics, who followed the vegetarian diet in urban cohort and mixed diet in rural cohort. In the urban cohort, 56.8%, 37% and 10% of participants had hypertension, diabetes and cardiac illness, respectively. Whereas, in the rural cohort, 33.3%, 27.7%, and 1.9% participants had hypertension, diabetes and cardiac illness, respectively.

**Table 1:** Comparison of demographic characteristics between urban and rural cohorts

Variables	Urban cohort (*n* = 923)	Rural cohort (*n* = 4239)	*P-*value
Age, median (IQR)	64 (14)	58 (15)	<0.001
Current gender, *n* (%)			
Female	447 (48.4)	2219 (52.3)	0.03
Male	476 (51.6)	2020 (47.7)	
Smoking, *n* (%)			
Never smoked	510 (55.3)	2251 (53.1)	<0.001
Ex-smokers	379 (41.1)	549 (13)	
Current smokers	34 (3.7)	1439 (33.9)	
Alcohol consumption, *n* (%)			
Non drinkers	784 (84.9)	3970 (93.7)	<0.001
Current drinkers	139 (15.1)	269 (6.3)	
Diet, *n* (%)			
Vegetarian	534 (57.9)	476 (11.2)	<0.001
Mixed diet	389 (42.1)	3763 (88.8)	
Hypertension, *n* (%)			
Yes	524 (56.8)	1410 (33.3)	<0.001
No	399 (43.2)	2829 (66.7)	
Diabetes, *n* (%)			
Yes	341 (37)	1175 (27.7)	<0.001
No	582 (63)	3064 (72.3)	
Cardiac illness, *n* (%)			
Yes	92 (10)	82 (1.9)	<0.001
No	831 (90)	4157 (98.1)	
HCY, median (IQR)	17.70 (10.2)	14.70 (9.7)	<0.001
Vitamin B12, median (IQR)	251.2 (231)	219 (138)	<0.001
Folic acid, median (IQR)	8.21 (8)	5.48 (4)	<0.001

Abbreviations: IQR, Interquartile range; HCY, Homocysteine. *P*-value < 0.05 was statistically significant.

There was a significant difference in HCY (median (IQR) 17.70 (10.2) versus 14.70 (9.7); *P* < 0.001), vitamin B12 (251 (231) versus 219 (138); *P* < 0.001) and folic acid (8.21 (8) versus 5.48 (4); *P* < 0.001) levels between urban and rural participants, with significantly higher levels in urban participants (Table 1). We also made an attempt in residualizing age by using the age-residualized variables in the non-parametric analyses. We found that the results remained unchanged (all *P* < 0.001).

In addition, urban–rural differences in HCY, vitamin B12 and folic acid were estimated separately for non-vegetarians and vegetarians using model 1 and model 2. HCY level in the urban cohort was found significantly more than the rural cohort among non-vegetarians [Model 1: *β* (95% CI) −3.51 (−4.45 to −2.56), *P* < 0.001; Model 2: *β* (95% CI) −2.79 (−3.77 to −1.82), *P* < 0.001], but it was found insignificant in its counterparts. However, vitamin B12 level was found significantly more in urban than the rural cohort among both vegetarians [Model 1: *β* (95% CI) −54.89 (−82.78 to −27.01), *P* < 0.001; Model 2: *β* (95% CI) −42.05 (−72.68 to −11.42), *P* = 0.007] and non-vegetarians [Model 1: *β* (95% CI) −105.65 (−125.03 to −86.28), *P* < 0.001; Model 2: *β* (95% CI) −89.56 (−110.73 to −68.39), *P* < 0.001]. Similarly folic acid was also higher in urban than rural both in vegetarians [Model 1: *β* (95% CI) −2.91 (−3.69 to −2.14), *P* < 0.001; Model 2: *β* (95% CI) −2.58 (−3.46 to −1.70), *P* < 0.001] and non-vegetarians [Model 1: *β* (95% CI) −3.33 (−3.92 to −2.74), *P* < 0.001; Model 2: *β* (95% CI) −2.97 (−3.58 to −2.36), *P* < 0.001].

Furthermore, we found that the choice of diet was a mediating factor between the HCY level and study cohorts wherein the indirect effect [OR (95% CI) 0.75 (0.69 to 0.82)] of the cohorts through the choice of diet on HCY level was 43% of the total effect (z = 2.67, *P* < 0.001).

Table 2 and Supplementary Fig. 1 show the gender-wise comparison of these levels. Significant gender difference was seen in HCY (males > females, *P* < 0.001), vitamin B12 (females > males, *P* < 0.001) and folic acid (females > males, *P* = 0.003) for the urban cohort. Similarly, gender difference was seen in HCY (males > females, *P* < 0.001) and folic acid (females > males, *P* < 0.001) for the rural cohort. There was no significant difference in vitamin B12 levels. Supplementary Table 1 shows the comparison of these levels across the age groups (45–54 versus 55–64 versus 65–74 versus ≥ 75 years). Significant difference in vitamin B12 (*P* < 0.001) and folic acid (*P* = 0.006) across the age group in TLSA and significant difference across the age group in HCY (*P* < 0.001) in SANSCOG were observed. The results remained consistent for the age-residualized variables (all *P* < 0.001), except for vitamin B12 in the rural cohort, (males > females, *P* = 0.003).

**Table 2:** Comparison of HCY, vitamin B12 and folic acid levels between females and males in urban and rural cohorts

Variables	Urban cohort		*P*-value	Cohen's d (95% CI)	Rural cohort		*P*-value	Cohen's d (95% CI)
	Female (*n* = 447)	Male (*n* = 476)			Female (*n* = 2219)	Male (*n* = 2020)		
	Median (IQR)				Median (IQR)			
HCY	15.40 (7.5)	20.60 (10.9)	<0.001	−0.61 (−0.74 to −0.48)	12.78 (7.9)	17.26 (11.7)	<0.001	−0.60 (−0.66 to −0.54)
Vitamin B12	268 (239)	231 (213)	<0.001	0.196 (0.07 to 0.35)	217 (142)	220 (132)	0.55	0.02 (−0.03 to 0.09)
Folic acid	9.18 (9)	7.48 (8)	0.003	0.21 (0.05 to 0.38)	5.86 (4)	5.06 (3)	<0.001	0.21 (0.15 to 0.27)

Abbreviations: IQR, Interquartile range; HCY, Homocysteine; CI, Confidence interval. *P*-value < 0.05 was statistically significant.

Within the urban cohort, 847 participants exhibited normal cognition, while 76 had MCI (CDR global score). Similarly, in rural cohort, 3886 had normal cognition, and 353 had MCI (CDR global score). HCY, vitamin B12 and folic acid levels did not differ significantly between normal and MCI participants for the overall sample, as well as across the age group in both the cohorts, when CDR global score was used to diagnose MCI (Table 3). Upon further analysis using CDR-SOB to diagnose MCI (Supplementary Table 2), no significant difference was found between normal and MCI participants in the urban cohort. However, in the rural cohort, significantly higher levels of HCY were observed in normal participants for the overall sample (Median (IQR) 14.95 (9.65) versus 14.23 (9.61); *P* = 0.03), as well as for <60 years (14.05 (9.23) versus 13.05 (7.85); *P* = 0.02) and ≥60 years of age (16.12 (10.43) versus 15.48 (10.84); *P* = 0.03), and higher levels of vitamin B12 (213 (118) versus 228 (149); *P* = 0.04) and folic acid (5.27 (4) versus 5.68 (4); *P* = 0.03) was observed in <60 years and ≥60 years of age, respectively, in MCI participants, with small effect size. To ensure statistical robustness, comparison was done between MCI and age-gender matched normal participants, and there was no significant difference observed in the urban cohort. In the rural cohort, participants with normal cognition had higher levels of vitamin B12 in those who are ≥60 years of age (227 (152) versus 217 (175); *P* = 0.03) and higher folic acid in those <60 years of age (5.91 (4) versus 5.40 (4); *P* = 0.04) compared to MCI (Supplementary Table 3).

**Table 3:** Comparison of HCY, vitamin B12 and folic acid levels between normal and MCI subjects (using CDR global score) urban and rural cohorts

Variables	Urban cohort		*P*-value	Cohen's d (95% CI)	Rural cohort		*P*-value	Cohen's d (95% CI)
	Normal (*n* = 847)	MCI (*n* = 76)			Normal (*n* = 3886)	MCI (*n* = 353)		
	Median (IQR)				Median (IQR)			
HCY								
All	17.45 (10.33)	19.30 (9.99)	0.06	−0.19 (−0.43 to 0.04)	14.75 (9.65)	14.37 (11.15)	0.13	0.06 (−0.05 to 0.17)
<60 years	17.82 (10.01)	20.46 (14.53)	0.37	−0.26 (−0.79 to 0.25)	13.84 (8.92)	12.89 (9.42)	0.15	0.05 (−0.14 to 0.25)
≥60 years	17.44 (10.29)	19.10 (9.92)	0.09	−0.18 (−0.44 to 0.08)	16 (10.41)	15.04 (11.62)	0.02	0.14 (0.01 to 0.28)
Vitamin B12								
All	251 (232)	268.5 (209)	0.40	0.004 (−0.23 to 0.24)	219 (135)	217 (172)	0.76	−0.11 (−0.22 to 0.00)
<60 years	224 (190)	254.50 (118)	0.51	0.08 (−0.45 to 0.62)	215 (122)	218 (151)	0.81	−0.08 (−0.28 to 0.12)
≥60 years	265 (260)	268.50 (224)	0.85	0.05 (−0.21 to 0.32)	222 (151)	216 (181)	0.88	−0.07 (−0.21 to 0.06)
Folic acid								
All	8.19 (8)	8.30 (9)	0.94	0.05 (−0.26 to 0.35)	5.49 (4)	5.28 (4)	0.32	0.06 (−0.05 to 0.18)
<60 years	7.52 (8)	6.73 (5)	0.12	0.43 (−0.24 to 1.09)	5.58 (4)	5 (3)	0.17	0.13 (−0.08 to 0.34)
≥60 years	9.09 (9)	8.58 (8)	0.82	0.04 (−0.31 to 0.38)	5.43 (4)	5.35 (4)	0.81	0.05 (−0.09 to 0.19)

Abbreviations: MCI, Mild cognitive impairment; IQR, Interquartile range; HCY, Homocysteine; CI, Confidence interval. *P*-value < 0.05 was statistically significant.

For the regression analysis, a fully adjusted model (i.e. Model 3) was used for interpretation. There was no significant association seen between HCY, vitamin B12 and MCI for combined as well as individual cohorts for overall sample and also across the age groups upon using CDR global score for diagnosing MCI (Supplementary Tables 4 and 5). However, when using CDR-SOB, there was no significant relationship between HCY and CDR-SOB (Supplementary Table 6). A significant positive relationship was found between vitamin B12 deficiency and CDR-SOB in rural cohort [*β* (95% CI) 0.05 (0.005 to 0.10); *P* = 0.03] and in overall sample of individuals [*β* (95% CI) 0.07 (0.02 to 0.11); *P* = 0.004] aged 60 years and above (Supplementary Table 7). Additionally, we found a significant positive relationship between folic acid deficiency and CDR-SOB in urban cohort of individuals <60 years of age [*β* (95% CI) 0.22 (0.08 to 0.36); *P* = 0.002] (Supplementary Table 8). Further, analysis was done to check the association between various combinations of HCY–vitamin B12 levels and MCI (using CDR global). However, no significant association was observed (Supplementary Table 9).

We also performed mediation analysis to check whether hypertension, diabetes, cardiac illness and FRS were the mediator between HCY and cognition (CDR-SOB). Hypertension, diabetes and cardiac illness did not have any mediating effects between HCY and cognition. However, we found that FRS had a mediating effect on cognition via HCY level only in the rural cohort [indirect effect *β* (95% bootstrap CI) 0.011 (0.006 to 0.016)]. Additionally, we also found that gender did not show a moderating effect between HCY and cognition (CDR-SOB).

Baseline characteristics of the participants with high HCY are described in the Supplementary Table 10. Participants with high HCY were above the age of 60, males, non-smokers, non-alcoholics and consumed mixed diet; 39.6% had hypertension (56.1% in urban, 34.8% in rural); 26.7% had diabetes (33.8% in urban, 24.7% in rural); 4% had cardiac illness (10.1% in urban, 2.2% in rural); 51.2% had vitamin B12 deficiency (43.8% in urban, 53.2% in rural); and 10.1% had folic acid deficiency (2.2% in urban, 12.5% in rural). The prevalence of MCI was 8.4% in overall sample (9.3% in urban, 8.2% in rural) when CDR global score was used to diagnose MCI. However, the prevalence has increased to 20.8% in overall sample (11.1% in urban, 23.7% in rural) when CDR-SOB was used for diagnosing MCI.

**Figure 2:**
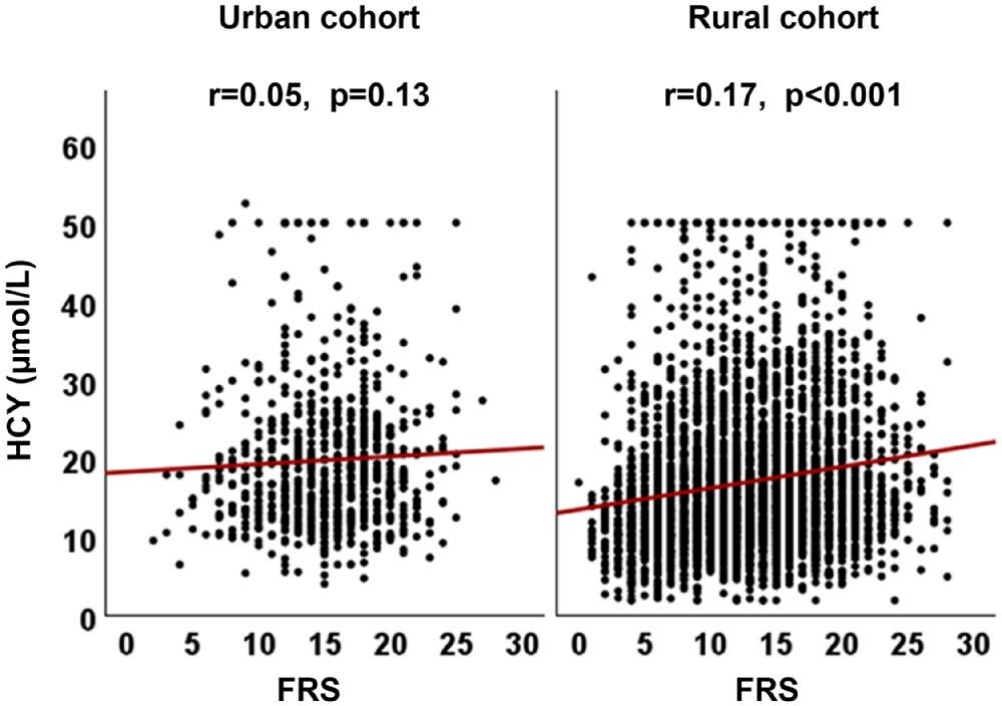
**HCY and FRS correlation.** Correlation between homocysteine (HCY) and Framingham risk score (FRS) in urban and rural cohorts. r-Spearman correlation coefficient.

There was no correlation between HCY and FRS in the urban cohort. A weak, but statistically significant correlation (r = 0.17, *P* < 0.001) was seen in the rural cohort (Fig. 2).

## Discussion

The present study aimed to explore the relationship between HCY, vitamin B12, folic acid levels and cognitive health in urban, as well as rural Indian populations. Further evaluation was done to see the association between HCY and FRS.

Relatively fewer studies have reported the comparison of HCY, vitamin B12 and folic acid levels between rural and urban populations. We observed higher HCY, vitamin B12 and folic acid levels in urban participants compared to their rural counterparts. This difference may be partially explained by the higher prevalence of non-vegetarian diet in the rural cohort, which could influence HCY levels. This could also be attributed to various factors, including environmental influences like air pollution. There is also emerging evidence on association of air pollution and elevated HCY levels.^16,17^ Despite the higher levels of vitamin B12 and folic acid observed in the urban cohort, both cohorts exhibited levels within the normal range. Several factors inherent to urban living may contribute to this finding. Urban areas typically offer better access to a diverse range of foods, including fortified products and dietary supplements. Moreover, the widespread availability of over-the-counter medicines, including vitamin supplements may contribute to increased intake of these nutrients among the urban residents. Furthermore, urban populations may demonstrate greater awareness and access to healthcare services, leading to more frequent health check-ups and interventions, such as periodic deworming, which can enhance nutrient absorption and metabolism. Additionally, socioeconomic disparities between urban and rural areas may also play a role.

Gender plays an important role in HCY, vitamin B12 and folic acid levels. Cross-sectional studies from China and Israel have shown the higher level of HCY in men than women.^18,19^ Few other studies have reported lower vitamin B12 and folic acid levels in men compared to women.^19-21^ Gender differences were evident in HCY, vitamin B12 and folic acid levels, with higher HCY levels in men in both urban and rural cohorts. Men had lower vitamin B12 and folic acid levels in the urban cohort and lower folic acid levels in rural cohort. Possible reasons for these differences include gender-specific variations in HCY metabolism and lifestyle practices like smoking^22^ and alcohol consumption.^23^

Interestingly, HCY levels did not show significant differences across age groups, but vitamin B12 and folic acid levels increased gradually with age in the urban cohort. This could be due to the higher level of education and better access to healthcare in urban areas, leading to more elderly individuals receiving vitamin B12 supplementation to address deficiencies or medical conditions that can cause low B12 levels. However, in the rural cohort, HCY levels increased with age with a decrease in vitamin B12 levels. This explains the inverse relationship between HCY and vitamin B12 or folic acid.^24^

In our urban cohort, we did not observe any significant difference in HCY, vitamin B12 and folic acid levels between normal and MCI participants. However, within the rural cohort, there was no difference in HCY levels, but lower levels of vitamin B12 and folic acid were seen in specific age groups among MCI participants when compared to age-gender matched normal participants. Some studies have found a significant association between elevated HCY levels, low vitamin B12 and folic acid levels and an increased risk of cognitive impairment.^25-28^ Meanwhile, others have found no association.^29-31^ Further, we found no significant association between HCY levels and cognitive status. However, there was a significant positive relationship between vitamin B12 deficiency and CDR-SOB, as well as folic acid deficiency and CDR-SOB in rural (≥60 years) and urban (<60 years) cohorts, respectively. Additionally, we did not find any association between various combinations of HCY-vitamin B12 levels and MCI, even after adjusting for various factors. Our finding is consistent with results from a previously reported Rotterdam study in the Netherlands, which concluded the lack of association between HCY and MCI.^32^ The Framingham Study, which evaluated the relation of the plasma total HCY level measured at baseline and its risk of developing dementia after a median follow-up after 8 years, concluded that an increased plasma HCY level is a strong, independent risk factor for the development of dementia.^25^ The Veterans Affairs Normative Aging Study revealed cognitive decline in association with low B vitamin and high HCY concentrations.^26^ The Leiden 85-Plus Study, a population-based longitudinal study of 599 participants in the Netherlands, showed that the elevated levels of HCY and reduced folic acid are associated with cognitive impairment.^28^ A study involving 1408 participants from the Boston Puerto Rican Health Study found that low plasma vitamin B12 and low plasma folate were each associated with poorer cognitive function.^33^ Periodic follow-up of participants in our cohort with cognitive assessments in the future may give a better understanding of causal relationship between elevated HCY, low vitamin B12, folic acid levels and cognitive impairment.

HCY and FRS are linked to cardiovascular diseases, but the correlation between the two is not well established. Some studies have shown an association between HCY levels and cardiovascular risk,^34,35^ while others have found no significant association. However, we observed no clear association between HCY and FRS in the urban cohort and a weak correlation in the rural cohort. Cardiovascular disease is a complex condition influenced by various factors, such as family history, obesity, diabetes and physical activity, beyond HCY and FRS. A comprehensive risk assessment is necessary, which will be addressed in our future longitudinal study with regular follow-up assessments.

Our study found that 51.2% of participants had vitamin B12 and folic acid deficiency in those with hyperhomocysteinemia, with notable difference between urban (43.8%) and rural (53.2%). Additionally, 10.1% of the participants had folic acid deficiency (2.2% in urban, 12.5% in rural) in those with hyperhomocysteinemia. This high prevalence of vitamin B12 and folic acid deficiencies in our study, particularly in the rural cohort, is noteworthy, even though percentage of non-vegetarians is higher (85.7%). This could be due to infrequent consumption of non-vegetarian foods, absorption-related issues, such as gastrointestinal disorders or parasitic infections, and traditional methods of cooking in rural areas, such as prolonged boiling or high-temperature cooking, which lead to significant nutrient loss.

The strengths of our study include a large sample size from both urban and rural regions, the comprehensive investigation of lifestyle factors, such as diet, and bioassays performed at a nationally accredited laboratory. However, our study has certain limitations, such as a non-representative sample, which restricts the generalizability of results, and the detail data on vitamin supplement intake leading to confounding bias. Additionally, low prevalence of MCI in our cohorts may have impeded our ability to detect the effect. Another limitation is lack of continuous cognitive measure, apart from CDR-SOB. Similar studies from other regions of India would address the limitations of sample representation.

## Conclusion

The present study compared the HCY, vitamin B12 and folic acid levels between urban and rural populations and their association with MCI. This study also attempts to correlate HCY with FRS. Urban participants had higher levels of HCY, vitamin B12 and folic acid. Gender differences were observed, with men having higher HCY and lower folate levels. This also shows the need for screening of essential vitamin deficiencies, especially in elderly population along with the necessary correction or preventive food fortification measures mainly in rural population. Age-related trends differed between urban and rural cohorts. In the rural cohort, participants with MCI had lower vitamin B12 and folic acid levels in a certain age group. There was no significant association between HCY and cognitive status, but there was a significant positive relationship between vitamin B12 deficiency and CDR-SOB, as well as folic acid deficiency and CDR-SOB in rural and urban cohorts, respectively, within a specific age group. Additionally, the correlation between HCY and FRS was weak in rural participants and absent in urban participants. These findings indicate the complex nature of metabolic risk factors and their impact on cognitive health. Future perspectives should probe into the underlying mechanism and potential intervention for prevention of dementia through longitudinal studies, considering lifestyle, genetics and environmental factors.

## Supplementary Material

fcae343_Supplementary_Data

## Data Availability

Data extracted from this study can be obtained from the corresponding author, LD, upon reasonable request.
