# Supplementary material for: Comparison of homocysteine, vitamin B12 and folic acid between rural and urban ageing Indians and its association with mild cognitive impairment and cardiovascular risk factors: a cross-sectional analysis
Source: Brain Commun. 2024 Oct 15;6(5):fcae343. doi: 10.1093/braincomms/fcae343 (PMC11474239; doi:10.1093/braincomms/fcae343)
Supplement: fcae343_Supplementary_Data [file fcae343_supplementary_data.docx]

**Supplementary material**

| Urban cohort | 45-54 years  (n=172) | 55-64 years  (n=315) | 65-74 years  (n=323) | ≥ 75 years  (n=113) | *p-*value | Multiple comparison^*^ |
| --- | --- | --- | --- | --- | --- | --- |
|  | **Median (IQR)** | | | |  |  |
| HCY | 17·79 (10.46) | 17·51 (11.06) | 17·73 (10.43) | 17·78 (9.76) | 0·98 | __ |
| Vitamin B12 | 225.50 (183) | 246 (200) | 267 (259) | 280 (433) | <0·001 | G1 vs G3 (p =0·001)  G1 vs G4 (p <0·001)  G2 vs G4 (p =0·005) |
| Folic acid | 6·92 (6) | 8·70 (7.01) | 8.76 (8) | 11·70 (10) | 0·006 | G1 vs G2 (p =0·007)  G1 vs G3 (p =0·003)  G1 vs G4 (p =0·009) |
| Rural cohort | **(n=1626)** | **(n=1294)** | **(n=1006)** | **(n=313)** |  |  |
| HCY | 13·66 (9) | 14·63 (9.48) | 15.94 (10.39) | 18 (12.70) | <0·001 | G1 vs G2 (p <0·001)  G1 vs G3 (p <0·001)  G1 vs G4 (p <0·001)  G3 vs G4 (p =0·004)  G2 vs G3 (p =0·003)  G2 vs G4 (p <0·001) |
| Vitamin B12 | 213 (119) | 221 (139) | 221 (153) | 226 (177) | 0·01 | G1 vs G2 (p =0·01) |
| Folic acid | 5·54 (4) | 5·46 (4) | 5·60 (4) | 5·11 (3) | 0·06 | __ |

**Supplementary Table 1: Comparison of HCY, Vitamin B12 & Folic acid among different age groups.**

Abbreviations: IQR – Interquartile range; HCY – Homocysteine; G1- 45-54 years; G2- 55-64 years; G3-65-74 years; G4- ≥ 75 years. *p*-value <0.05 was statistically significant. ^*^Bonferroni corrected *p*-value for multiple comparison, *p*-value < 0.0125 (0.05/4) was statistically significant.

**Supplementary Table 2:** **Comparison of HCY, vitamin B12, and folic acid levels between normal and MCI participants (using CDR-SOB) in urban and rural cohorts.**

| Variables | Urban cohort | | *p*-value | Cohen’s d  (95% CI) | Rural cohort | | *p*-value | Cohen’s d  (95% CI) |
| --- | --- | --- | --- | --- | --- | --- | --- | --- |
|  | **Normal (n=828)** | **MCI (n=95)** |  |  | **Normal (n=3172)** | **MCI (n=1067)** |  |  |
|  | **Median (IQR)** | |  |  | **Median (IQR)** | |  |  |
| HCY  All | 17·44 (10·28) | 19·35 (10.40) | 0·06 | -0.21  (-0.42 to 0.00) | 14·95 (9·65) | 14·23 (9·61) | 0·03 | 0.09  (0.02 to 0.16) |
| <60 years | 17.79 (10.13) | 20.19 (10.53) | 0.41 | -0.15  (-0.57 to 0.27) | 14.05 (9.23) | 13.05 (7.85) | 0.02 | 0.13  (0.02 to 0.23) |
| ≥60 years | 17.44 (10.15) | 19.10 (10.41) | 0.09 | -0.23  (-0.48 to 0.01) | 16.12 (10.43) | 15.48 (10.84) | 0.03 | 0.13  (0.03 to 0.22) |
| Vitamin B12  All | 252.25 (232) | 247 (235) | 0·67 | 0.04  (-0.17 to 0.25) | 218 (127) | 222 (164) | 0·63 | -0.05  (-0.12 to 0.02) |
| <60 years | 224.50 (191) | 227 (160) | 0.68 | 0.23  (-0.19 to 0.66) | 213(118) | 228 (149) | 0.04 | -0.11  (-0.21 to -0.01) |
| ≥60 years | 266 (257) | 256.50 (243) | 0.61 | 0.04  (-0.21 to 0.28) | 222 (147) | 217 (179) | 0.12 | 0.02  (-0.07 to 0.11) |
| Folic acid  All | 8·17 (8) | 8·39 (8) | 0·65 | -0.04  (-0.31 to 0.23) | 5·43 (4) | 5·60 (4) | 0·23 | -0.06  (-0.13 to 0.02) |
| <60 years | 7.52 (7) | 6.10 (6) | 0.68 | 0.003  (-0.50 to0.51) | 5.56 (4) | 5.40 (4) | 0.59 | 0.004  (-0.11 to 0.12) |
| ≥60 years | 8.98 (8) | 8.91 (8) | 0.78 | 0.01  (-0.31 to 0.33) | 5.27 (4) | 5.68 (4) | 0.03 | -0.10  (-0.12 to 0.00) |

Abbreviations: MCI- Mild Cognitive Impairment; IQR – Interquartile range; HCY – Homocysteine; CI- Confidence Interval. *p*-value <0.05 was statistically significant.

**Supplementary Table 3:** **Comparison of HCY, vitamin B12, and folic acid levels between matched MCI-normal participants (using CDR-SOB) in urban and rural cohorts**

| Variables | Urban cohort | | *p*-value | Cohen’s d  (95% CI) | Rural cohort | | *p*-value | Cohen’s d  (95% CI) |
| --- | --- | --- | --- | --- | --- | --- | --- | --- |
|  | **Normal (n=94)** | **MCI (n=94)** |  |  | **Normal (n=1044)** | **MCI (n=1044)** |  |  |
|  | **Median (IQR)** | |  |  | **Median (IQR)** | |  |  |
| HCY  All | 18·37 (10·46) | 19·37 (10.45) | 0·29 | -0.18  (-0.46 to 0.11) | 14·19 (8.48) | 14·18 (9·60) | 0·61 | -0.03  (0.12 to 0.05) |
| <60 years | 17.22 (15.42) | 20.19 (10.53) | 0.59 | -0.06  (-0.64 to 0.51) | 13.06 (8.50) | 13.05 (7.85) | 0.90 | -0.01  (-0.14 to 0.12) |
| ≥60 years | 18.67 (9.99) | 19.35 (10.44) | 0.40 | -0.21  (-0.54 to 0.12) | 14.88 (8.72) | 15.30 (10.84) | 0.35 | -0.05  (-0.16 to 0.06) |
| Vitamin B12  All | 277  (252) | 248  (238) | 0·49 | 0.09  (-0.19 to 0.38) | 222 (133) | 222 (161) | 0·35 | 0.01  (-0.08 to 0.09) |
| <60 years | 269  (263) | 227  (160) | 0.34 | 0.51  (-0.07 to 1.10) | 217(115) | 228 (149) | 0.21 | -0.09  (-0.22 to 0.04) |
| ≥60 years | 282  (276) | 260  (243) | 0.80 | 0.02  (-0.31 to 0.34) | 227 (152) | 217 (175) | 0.03 | 0.07  (-0.04 to 0.18) |
| Folic acid  All | 10.85 (9) | 8·39 (8) | 0·61 | 0.18  (-0.22 to 0.57) | 5·70 (4) | 5·60 (4) | 0·83 | -0.01  (-0.10 to 0.08) |
| <60 years | 10.28 (9) | 6.10 (6) | 0.25 | 0.38  (-0.37 to 1.14) | 5.91 (4) | 5.40 (4) | 0.04 | 0.12  (-0.02 to 0.26) |
| ≥60 years | 10.85 (9) | 8.91 (8) | 0.88 | 0.11  (-0.35 to 0.58) | 5.39 (4) | 5.71 (4) | 0.12 | -0.10  (-0.22 to 0.02) |

Abbreviations: MCI- Mild Cognitive Impairment; IQR – Interquartile range; HCY – Homocysteine**;** CI- Confidence Interval. *p*-value <0.05 was statistically significant.

| Variables |  | Model 1 | | Model 2 | | Model 3 | |
| --- | --- | --- | --- | --- | --- | --- | --- |
|  |  | **OR (95% CI)** | ***p-*value** | **OR (95% CI)** | ***p-*value** | **OR (95% CI)** | ***p-*value** |
| HCY  (>15μmol/L) | **Urban cohort** |  |  |  |  |  |  |
|  | Combined | 1·54 (0·90-2·61) | 0·11 | 1.46 (0·83-2.58) | 0.19 | 0.97 (0.45-2.12) | 0.94 |
|  | <60 years | 1.29 (0.39-4.23) | 0.67 | 1.21 (0.34-4.27) | 0.77 | 0.66 (0.12-3.58) | 0.79 |
|  | ≥ 60 years | 1.63 (0.89-2.95) | 0.11 | 1.55 (0.81-2.95) | 0.18 | 1.08 (0.44-2.66) | 0.88 |
|  | **Rural cohort** |  |  |  |  |  |  |
|  | Combined | 0·96 (0·77-1.19) | 0.73 | 0·88 (0·69-1·12) | 0·29 | 0·86 (0·66-1·12) | 0·25 |
|  | <60 years | 0.92 (0.61 -1.38) | 0.69 | 1.05 (0.69-1.62) | 0.81 | 1.03 (0.64-1.66) | 0.90 |
|  | ≥ 60 years | 0.83 (0.64-1.08) | 0.16 | 0.83 (0.63-1.10) | 0.18 | 0.79 (0.58-1.09) | 0.16 |
|  | **Overall** |  |  |  |  |  |  |
|  | Combined | 1.03 (0·85-1.26) | 0·75 | 0.95 (0·77-1.18) | 0·65 | 0·87 (0·68-1·11) | 0·27 |
|  | <60 years | 0.95 (0.65-1.39) | 0.81 | 1.10 (0.74-1.64) | 0.26 | 1.03 (0.66-1.62) | 0.89 |
|  | ≥ 60 years | 0.92 (0.72-1.17) | 0.49 | 0.91 (0.71-1.16) | 0.44 | 0.82 (0.61-1.10) | 0.18 |
| HCY  (≤ 15μmol/L ) | | 1·00 (Ref) | | 1·00 (Ref) | | 1·00 (Ref) | |

**Supplementary Table 4: Association of HCY and Mild Cognitive Impairment (using CDR global score)**

Abbreviations: HCY – Homocysteine**;** OR- Odds Ratio; CI-Confidence interval; Ref – Reference; *p*-value <0.05 was statistically significant.

Model 1- Unadjusted, Model 2- Adjusted for age, gender, smoking, alcohol consumption, diet, Hypertension, cardiac illness, diabetes, and Model 3 - Adjusted variables in model 2 plus vitamin B12, and folic acid.

**Supplementary Table 5: Association of Vitamin B12 and Mild Cognitive Impairment (using CDR global score)**

| Variables |  | Model 1 | | Model 2 | | Model 3 | |
| --- | --- | --- | --- | --- | --- | --- | --- |
|  |  | **OR (95% CI)** | ***p-*value** | **OR (95% CI)** | ***p-*value** | **OR (95% CI)** | ***p-*value** |
| Vitamin B12  (<200 pg/mL ) | **Urban cohort** |  |  |  |  |  |  |
|  | Combined | 1·14 (0·69-1.89) | 0·61 | 0.92 (0·53-1.58) | 0.76 | 0.81 (0.37-1.77) | 0.59 |
|  | <60 years | 1.72 (0.53-5.62) | 0.37 | 1.48 (0.42-5.19) | 0.54 | 1.55 (0.24-6.79) | 0.64 |
|  | ≥ 60 years | 0.94 (0.54-1.66) | 0.84 | 0.82 (0.44-1.52) | 0.53 | 0.64 (0.25-1.62) | 0.35 |
|  | **Rural cohort** |  |  |  |  |  |  |
|  | Combined | 1·01 (0·81-1.26) | 0.92 | 0·98 (0·78-1·23) | 0·85 | 0·84 (0·65-1·09) | 0·19 |
|  | <60 years | 1.19 (0.79 -1.81) | 0.40 | 1.19 (0.78-1.82) | 0.42 | 1.17 (0.74-1.85) | 0.51 |
|  | ≥ 60 years | 0.92 (0.70-1.20) | 0.54 | 0.89 (0.67-1.17) | 0.41 | 0.72 (0.53-1.45) | 0.40 |
|  | **Overall** |  |  |  |  |  |  |
|  | Combined | 1.03 (0·84-1.26) | 0·77 | 0.95 (0·77-1.17) | 0·63 | 0·82 (0·65-1·04) | 0·11 |
|  | <60 years | 1.24 (0.84-1.84) | 0.27 | 1.18 (0.79-1.76) | 0.26 | 1.19 (0.76-1.84) | 0.45 |
|  | ≥ 60 years | 0.91 (0.72-1.62) | 0.46 | 0.86 (0.67-1.10) | 0.24 | 0.70 (0.53-1.93) | 0.11 |
| Vitamin B12  ( ≥ 200 pg/mL ) | | 1·00 (Ref) | | 1·00 (Ref) | | 1·00 (Ref) | |

Abbreviations: OR- Odds Ratio; CI-Confidence interval; Ref – Reference. *p*-value <0.05 was statistically significant.

Model 1- Unadjusted, Model 2- Adjusted for age, gender, smoking, alcohol consumption, diet, Hypertension, cardiac illness, diabetes, and Model 3 - Adjusted variables in model 2 plus vitamin B12, and folic acid.

| Variables | | Model 1 | | Model 2 | | Model 3 | |
| --- | --- | --- | --- | --- | --- | --- | --- |
|  |  | **β (95% CI)** | ***p-*value** | **β (95% CI)** | ***p-*value** | **β (95% CI)** | ***p-*value** |
| HCY  (>15μmol/L) | **Urban cohort** |  |  |  |  |  |  |
|  | Combined | 0.17 (-0.03 to 0.06) | 0·47 | 0.01 (-0.03 to 0.06) | 0.50 | 0.006 (-0.06 to 0.07) | 0.86 |
|  | <60 years | -0.02 (-0.07 to 0.02) | 0.38 | -0.01 (-0.06 to 0.03) | 0.59 | -0.05 (-0.11 to 0.12) | 0.14 |
|  | ≥ 60 years | 0.03 (-0.02 to 0.09) | 0.25 | 0.03 (-0.03 to 0.09) | 0.35 | 0.05 (-0.06 to 0.16) | 0.36 |
|  | **Rural cohort** |  |  |  |  |  |  |
|  | Combined | -0·01 (-0.03 to 0.02) | 0.48 | 0·001 (-0.02 to 0.03) | 0·96 | 0·002 (-0.03 to 0.03) | 0·91 |
|  | <60 years | -0.02 (-0.05 to 0.003) | 0.08 | 0.004 (-0.03 to 0.03) | 0.97 | 0.003 (-0.03 to 0.04) | 0.84 |
|  | ≥ 60 years | -0.02 (-0.06 to 0.02) | 0.36 | 0.002 (-0.04 to 0.05) | 0.93 | 0.97 (-0.05 to 0.05) | 0.99 |
|  | **Overall** |  |  |  |  |  |  |
|  | Combined | -0.01 (-0.04 to 0.01) | 0·17 | -0.001 (-0.02 to 0.02) | 0·93 | -0·002 (-0.03 to 0.03) | 0·90 |
|  | <60 years | -0.03(-0.05 to -0.01) | 0.01 | -0.01 (-0.03 to 0.02) | 0.66 | -0.006 (-0.03 to 0.02) | 0.67 |
|  | ≥ 60 years | -0.02 (-0.05 to 0.02) | 0.29 | 0.002 (-0.03 to 0.04) | 0.92 | 0.003 (-0.04 to 0.05) | 0.90 |
| HCY  (≤ 15μmol/L ) | | 1·00 (Ref) | | 1·00 (Ref) | | 1·00 (Ref) | |

**Supplementary Table 6:** **Association between HCY and cognition (CDR –SOB)**

Abbreviations: HCY – Homocysteine**;** β – Regression coefficient; CI-Confidence interval; Ref – Reference. *p*-value <0.05 was statistically significant.

Model 1- Unadjusted, Model 2- Adjuste*d* for age, gender, smoking, alcohol consumption, diet, Hypertension, cardiac illness, diabetes, and Model 3 - Adjusted variables in model 2 plus vitamin B12, and folic acid.

**Supplementary Table 7:** **Association between Vitamin B12 and cognition (CDR –SOB)**

| Variables | | Model 1 | | Model 2 | | Model 3 | |
| --- | --- | --- | --- | --- | --- | --- | --- |
|  |  | **β (95% CI)** | ***p-*value** | **β (95% CI)** | ***p-*value** | **β (95% CI)** | ***p-*value** |
| Vitamin B12  (<200 pg/mL ) | **Urban cohort** |  |  |  |  |  |  |
|  | Combined | 0.007 (-0.03 to 0.05) | 0·73 | 0.03 (-0.02 to 0.07) | 0.22 | 0.03 (-0.03 to 0.10) | 0·33 |
|  | <60 years | -0.02 (-0.06 to 0.02) | 0.36 | -0.01 (-0.06 to 0.03) | 0.52 | -0.02 (-0.08 to 0.04) | 0.50 |
|  | ≥ 60 years | 0.03 (-0.03 to 0.09) | 0.34 | 0.04 (-0.02 to 0.11) | 0.17 | 0.07 (-0.04 to 0.18) | 0.23 |
|  | **Rural cohort** |  |  |  |  |  |  |
|  | Combined | 0·005 (-0.02 to 0.03) | 0.69 | 0·01 (-0.01 to 0.04) | 0·32 | 0·01 (-0.01 to 0.04) | 0.32 |
|  | <60 years | -0.02 (-0.04 to 0.009) | 0.20 | -0.01 (-0.04 to 0.02) | 0.41 | -0.02 (-0.05 to 0.01) | 0.17 |
|  | ≥ 60 years | 0.03 (-0.01 to 0.08) | 0.13 | 0.04 (-0.002 to 0.08) | 0.06 | 0.05 (0.005 to 0.10) | 0.03 |
|  | **Overall** |  |  |  |  |  |  |
|  | Combined | 0.009 (-0.01 to 0.03) | 0·42 | 0.02 (0.0003 to 0.04) | 0·05 | 0.02 (-0.004 to 0.05) | 0.09 |
|  | <60 years | -0.02 (-0.04 to 0.006) | 0.15 | -0.008 (-0.03 to 0.02) | 0.51 | -0.02 (-0.04 to 0.01) | 0.21 |
|  | ≥ 60 years | 0.04 (0.005 to 0.08) | 0.03 | 0.05 (0.02 to 0.09) | 0.004 | 0.07 (0.02 to 0.11) | 0.004 |
| Vitamin B12  ( ≥ 200 pg/mL ) | | 1·00 (Ref) | | 1·00 (Ref) | | 1·00 (Ref) | |

Abbreviations: β – Regression coefficient; CI-Confidence interval; Ref – Reference. *p*-value <0.05 was statistically significant.

Model 1- Unadjusted, Model 2- Adjusted for age, gender, smoking, alcohol consumption, diet, Hypertension, cardiac illness, diabetes, and Model 3 - Adjusted variables in model 2 plus vitamin B12, and folic acid.

**Supplementary Table 8:** **Association between Folic acid and cognition (CDR –SOB)**

| Variables | | Model 1 | | Model 2 | | Model 3 | |
| --- | --- | --- | --- | --- | --- | --- | --- |
|  |  | **β (95% CI)** | ***p-*value** | **β (95% CI)** | ***p-*value** | **β (95% CI)** | ***p-*value** |
| Folic acid  (<3 ng/mL ) | **Urban cohort** |  |  |  |  |  |  |
|  | Combined | 0.05 (-0.11 to 0.22) | 0·53 | 0.07 (-0.09 to 0.24) | 0.38 | 0.08 (-0.08 to 0.24) | 0·35 |
|  | <60 years | 0.18 (0.04 to 0.32) | 0.01 | 0.22 (0.08 to 0.35) | 0.002 | 0.22 (0.08 to 0.36) | 0.002 |
|  | ≥ 60 years | -0.06 (-0.34 to 0.22) | 0.67 | -0.06 (-0.34 to 0.21) | 0.64 | -0.04 (-0.31 to 0.24) | 0.79 |
|  | **Rural cohort** |  |  |  |  |  |  |
|  | Combined | -0.04 (-0.08 to 0.009) | 0.11 | -0·03 (-0.08 to 0.01) | 0·13 | -0·04 (-0.08 to 0.008) | 0.10 |
|  | <60 years | -0.01 (-0.06 to 0.04) | 0.64 | -0.009 (-0.06 to 0.04) | 0.71 | -0.02 (-0.07 to 0.03) | 0.50 |
|  | ≥ 60 years | -0.07 (-0.14 to 0.01) | 0.09 | -0.06 (-0.14 to 0.01) | 0.12 | -0.06 (-0.14 to 0.02) | 0.13 |
|  | **Overall** |  |  |  |  |  |  |
|  | Combined | -0.02 (-0.07 to 0.02) | 0·29 | -0.03 (-0.07 to 0.02) | 0·21 | -0.03 (-0.07 to 0.01) | 0·17 |
|  | <60 years | 0.003 (-0.04 to 0.05) | 0.91 | 0.002 (-0.04 to 0.05) | 0.93 | -0.004 (-0.05 to 0.04) | 0.88 |
|  | ≥ 60 years | -0.05 (-0.12 to 0.02) | 0.17 | -0.06 (-0.13 to 0.01) | 0.11 | -0.06 (-0.13 to 0.01) | 0.11 |
| Folic acid  ( ≥ 3 ng/mL ) | | 1·00 (Ref) | | 1·00 (Ref) | | 1·00 (Ref) | |

Abbreviations: β – Regression coefficient; CI-Confidence interval; Ref – Reference. *p*-value <0.05 was statistically significant.

Model 1- Unadjusted, Model 2- Adjusted for age, gender, smoking, alcohol consumption, diet, Hypertension, cardiac illness, diabetes, and Model 3 - Adjusted variables in model 2 plus vitamin B12, and folic acid.

**Supplementary Table 9: Association between various combinations of HCY-vitamin B12 and Mild cognitive impairment (CDR global).**

| Variables | | Model 1 |  | Model 2 |  | Model 3 |  |
| --- | --- | --- | --- | --- | --- | --- | --- |
|  |  | **OR (95% CI)** | ***p-*value** | **OR (95% CI)** | ***p-*value** | **OR (95% CI)** | ***p-*value** |
| Urban | High HCY, High VitB12 | 1.27 (0.73-2.19) | 0.40 | 1.28 (0.72-2.29) | 0.40 | 0.74 (0.34 – 1.06) | 0.45 |
|  | High HCY, Low VitB12 | 1.42 (0.80-2.50) | 0.23 | 1.76 (0.95-3.26) | 0.07 | 1.75 (0.86 – 3.59) | 0.12 |
|  | Low HCY, Low VitB12 | 1.13 (0.41-3.13) | 0.81 | 1.62 (0.56-4.70) | 0.37 | 1.52 (0.43 – 5.35) | 0.51 |
|  | Low HCY, High VitB12 | 1·00 (Ref) |  | 1·00 (Ref) |  | 1·00 (Ref) |  |
| Rural | High HCY, High VitB12 | 0.78 (0.64-0.93) | 0.01 | 0.86 (0.71 -1.05) | 0.14 | 0.86 (0.70 – 1.07) | 0.17 |
|  | High HCY, Low VitB12 | 0.88 (0.74-1.05) | 0.16 | 1.01 (0.84-1.22) | 0.89 | 0.94 (0.76 – 1.16) | 0.58 |
|  | Low HCY, Low VitB12 | 0.90 (0.73-1.12) | 0.35 | 0.92 (0.73-1.15) | 0.44 | 0.91 (0.72 – 1.17) | 0.47 |
|  | Low HCY, High VitB12 | 1·00 (Ref) |  | 1·00 (Ref) |  | 1·00 (Ref) |  |
| Overall | High HCY, High VitB12 | 0.74 (0.62-0.88) | 0.001 | 0.82 (0.68-0.98) | 0.03 | 0.86 (0.71 -1.05) | 0.14 |
|  | High HCY, Low VitB12 | 0.88 (0.75-1.04) | 0.14 | 1.06 (0.89-1.27) | 0.50 | 1.01 (0.84 - 1.22) | 0.88 |
|  | Low HCY, Low VitB12 | 0.97 (0.79-1.19) | 0.78 | 0.99 (0.79-1.23) | 0.92 | 0.92 (0.73 - 1.15) | 0.44 |
|  | Low HCY, High VitB12 | 1·00 (Ref) |  | 1·00 (Ref) |  | 1·00 (Ref) |  |

Abbreviations: HCY – Homocysteine**;** OR- Odds Ratio; CI-Confidence interval; Ref – Reference. *p*-value <0.05 was statistically significant.

Model 1- Unadjusted, Model 2- Adjusted for age, gender, smoking, alcohol consumption, diet, Hypertension, cardiac illness, diabetes, and Model 3 - Adjusted variables in model 2 plus vitamin B12, and folic acid.

**Supplementary Table 10:** **Demographic characteristics of participants with HCY >15μmol/L**

| Variables | Overall  (n=2670) | Urban cohort (n=603) | Rural cohort  (n=2067) |
| --- | --- | --- | --- |
| Age, median (IQR) | 60 (16) | 64 (13) | 60 (15) |
| Current gender, n (%) |  |  |  |
| Female | 1057 (39·6) | 238 (39·5) | 819 (39·6) |
| Male | 1613 (60·4) | 365 (60·5) | 1248 (60·4) |
| Smoking, n (%) |  |  |  |
| Never smoked | 1324 (49·6) | 320 (53.1) | 1004 (48·6) |
| Ex-smokers | 542 (20·3) | 255 (42.3) | 287 (13·9) |
| Current smokers | 804 (30.1) | 28 (4·6) | 776 (37·5) |
| Alcohol consumption, n (%) |  |  |  |
| Non drinkers | 2372 (88.8) | 498 (82·6) | 1874 (90·7) |
| Current drinkers | 298 (11.2) | 105 (17·4) | 193 (9·3) |
| Diet, n (%) |  |  |  |
| Vegetarian | 629 (23·6) | 270 (44.8) | 296 (14·3) |
| Mixed diet | 2041 (76·4) | 333 (55.2) | 1771 (85·7) |
| Hypertension, n (%) | 1058 (39·6) | 338 (56.1) | 720 (34.8) |
| Diabetes, n (%) | 714 (26·7) | 204 (33·8) | 510 (24·7) |
| Cardiac illness, n (%) | 107 (4) | 61 (10·1) | 46 (2·2) |
| Vitamin B12 (0-199·9), n (%) | 1364 (51·2) | 264 (43·8) | 1100 (53·2) |
| Folic acid (0-2.9), n (%) | 271 (10·1) | 13 (2·2) | 258 (12·5) |
| MCI (CDR =0·5)*  MCI (CDR 0.5 to 4)^a^ | 225 (8·4)  556 (20.8) | 56 (9·3)  67 (11.1) | 169 (8·2)  489 (23.7) |

Abbreviations: IQR – Interquartile range; HCY – Homocysteine**;** MCI – Mild Cognitive Impairment. *MCI diagnosis using Clinical Dementia Rating global score. ^a^ MCI diagnosis using Clinical Dementia Rating sum of the boxes.

**Supplementary Figure**


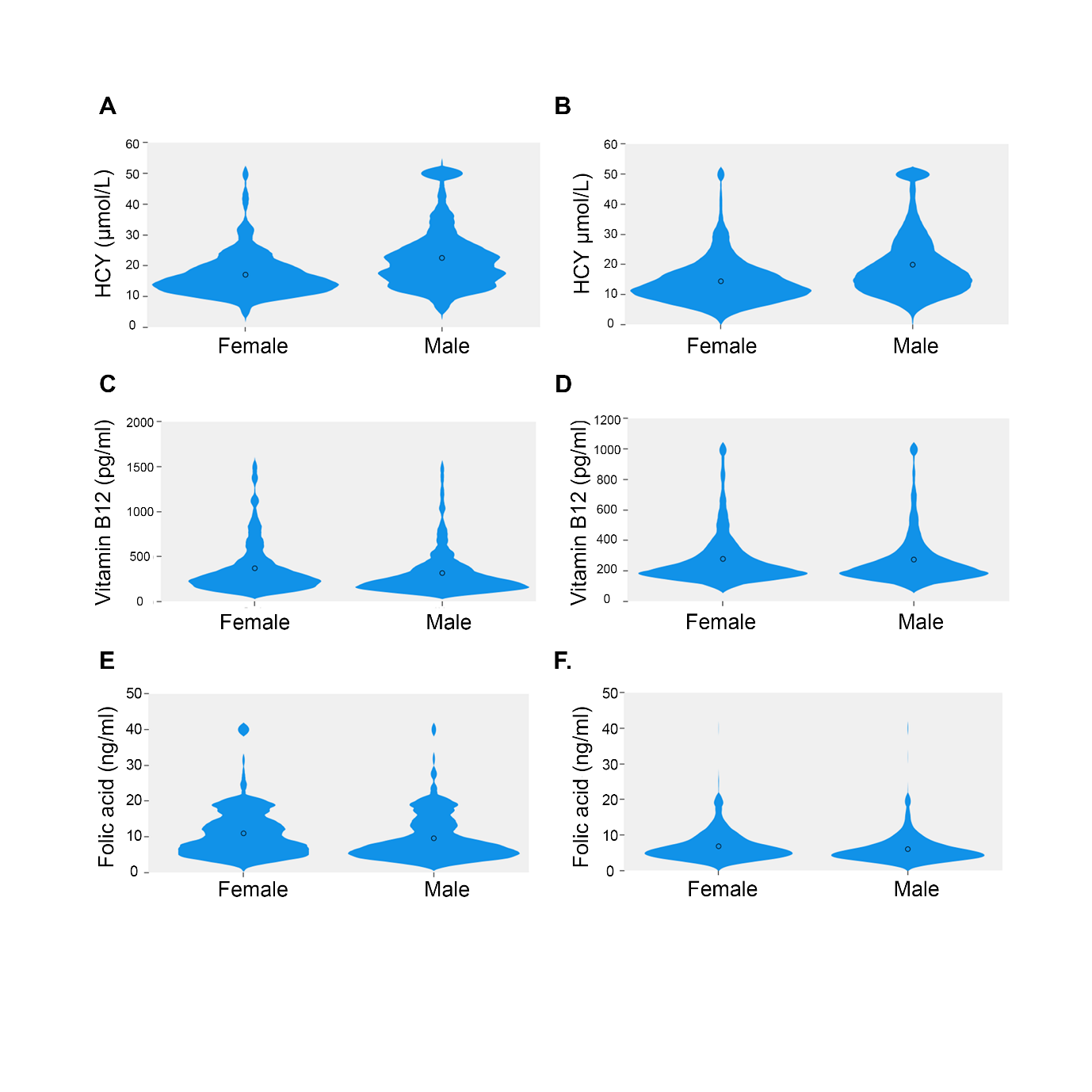


**Supplementary Figure 1:** Comparison of Homocysteine (HCY), vitamin B12 and folic acid levels between the genders within the cohort [Mann-Whitney U test]. A) HCY-urban cohort, [U=68064, Z=-9.47; *p* <0.001] B) HCY- rural cohort, [U=1464098, Z= -19.53; *p* <0.001] C) vitamin B12-urban cohort, [U=90959, Z= -3.81; *p* <0.001] D) vitamin B12-rural cohort, [U=2217448, Z=-0.60; *p* =0.55] E) folic acid – urban cohort, [U=33730, Z= -2.98; *p* =0.003] F) folic acid – rural cohort, [U=1483878, Z= -7.82; *p* <0.001].

**Short title: Gender difference in HCY, vitamin B12 and folic acid.**
